# Case report: Orbital myeloid sarcoma: a report of two rare cases and review of the literature

**DOI:** 10.3389/pore.2024.1611818

**Published:** 2024-10-25

**Authors:** Yanxi Li, Yujiao Wang, Weimin He

**Affiliations:** Department of Ophthalmology, West China Hospital of Sichuan University, Chengdu, Sichuan, China

**Keywords:** myeloid sarcoma (MS), orbit, histopathology, immunohistochemistry, diagnosis, treatment

## Abstract

Myeloid sarcoma (MS) occurs when primitive or naive myeloid cells form outside the bone marrow. It occurs mainly in soft/connective tissue and skin; orbital involvement is rare. We report the cases of two female adults, analyze the clinicopathologic characteristics, and review the literature. The average age of both patients was 28 years and they presented unilateral proptosis combined with varying degrees of impaired visual acuity and restricted ocular motility in the affected eye. Despite this, they maintained good overall health and no notable family history. However, the patients had no systemic clinical manifestations of acute myeloid leukemia (AML). Both patients underwent surgical resection of the orbital tumor. Immunohistochemistry showed positive staining for CD43, Leukocyte Common Antigen (LCA), and myeloperoxidase (MPO) and a high level of positive staining for Ki67, which were diagnostic for MS. Bone marrow cytology examination showed no apparent abnormalities. Postoperative chemotherapy, local radiotherapy, and allogeneic hematopoietic stem cell transplantation (allo-HSCT) were performed in Case 1, while the second patient underwent adjuvant chemotherapy and radiotherapy. No recurrence or metastasis was found in either patient during follow-up (one more than 5 years, the other more than 10 years). The occurrence of orbital MS is infrequent, with atypical clinical and imaging findings. The diagnosis depends on pathomorphology and immunohistochemical staining, and the prognosis is good with postoperative adjuvant chemotherapy, local radiotherapy, and allo-HSCT.

## Introduction

Myeloid diseases are a class of disorders involving the bone marrow and its hematopoietic functions, among which Myeloid Sarcoma (MS) represents a rare and high-grade hematological malignancy [1, 2]. As a special extramedullary manifestation of acute myeloid leukemia (AML), MS is characterized by the formation of extramedullary tumor masses composed of mature or immature myeloid neoplastic cells, which can disrupt the normal tissue structures, and its incidence is only 1.4% of AML cases [3, 4]. Because the clinical and imaging findings of orbital MS are atypical, ophthalmologists are confused by its diagnosis. In the present study, two cases of orbital MS are reported, highlighting their clinicopathological features and reviewing the relevant literature to provide more evidence for clinical treatment and pathological diagnosis.

## Case presentation

Two patients with orbital MS treated at West China Hospital of Sichuan University between 2011 and 2022 were identified by querying the Ophthalmology Department database. The clinical histories, manifestations, and pathologic findings associated with the tumors were retrospective investigations. A literature review on orbital MS was performed using PubMed and Web of Science.

### Case 1

**FIGURE 1:**
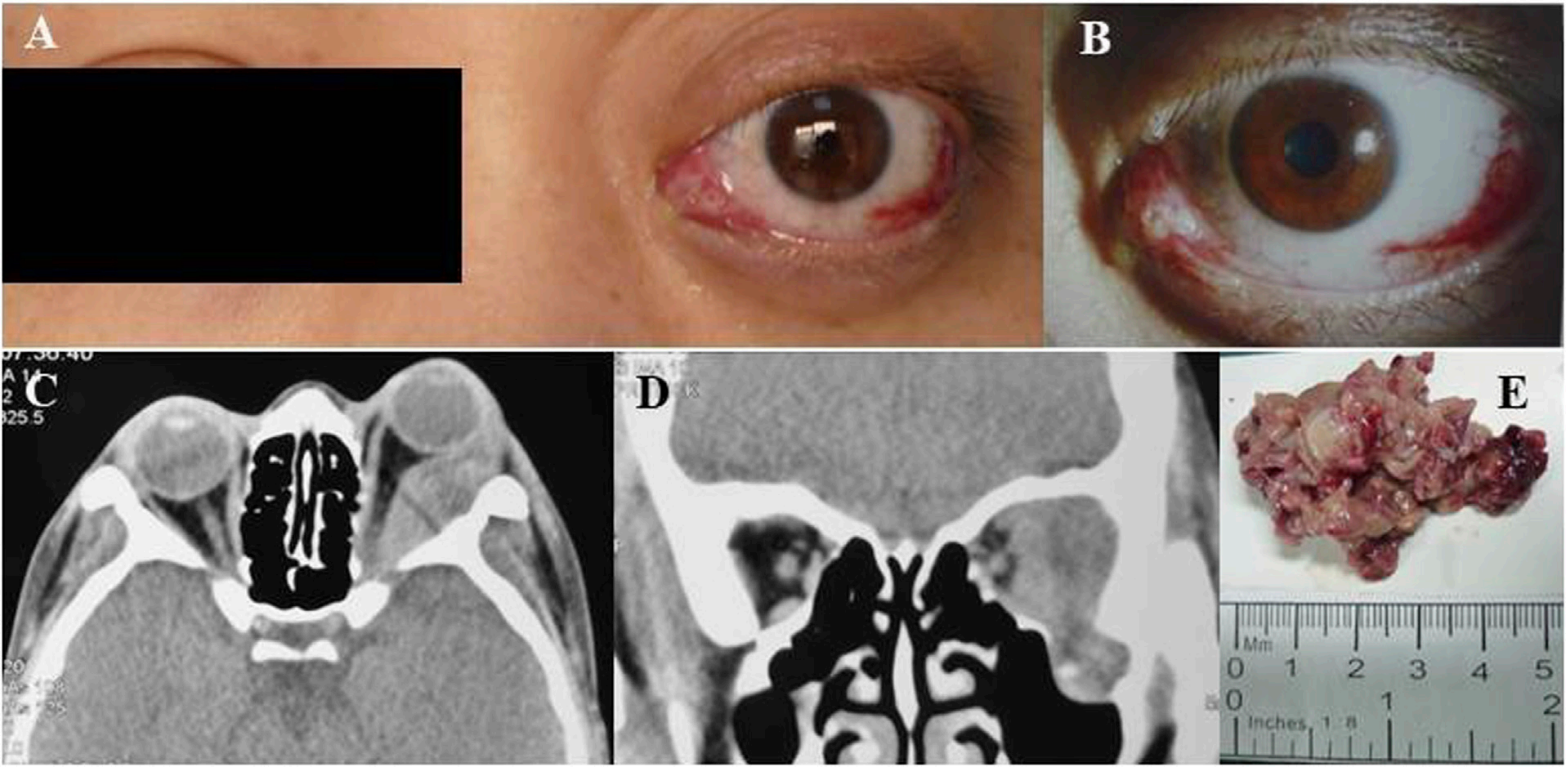
**(A)** Left preoptic bulge. **(B)** Left eye showing conjunctival congestion, edema and exophthalmos. **(C)** Axial CT scan of the orbits showing irregular mass in the left orbit with compression of the left orbital bulb, external rectus muscle and optic nerve nudged. **(D)** Coronal CT image revealed bone destruction was seen below the left eye and a partial mass invaded the temporal fossa. **(E)** orbital mass resected from patient 1.

**FIGURE 2:**
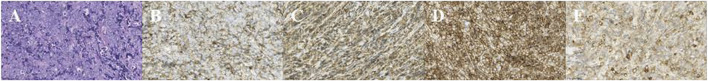
**(A)** HE, hematoxylin–eosin stain × 50; **(B)** LCA, Leukocyte Common Antigen × 50; **(C)** MPO, myeloperoxidase positivity × 50; **(D)** CD43 × 50; **(E)** CD117 × 50.

**FIGURE 3:**
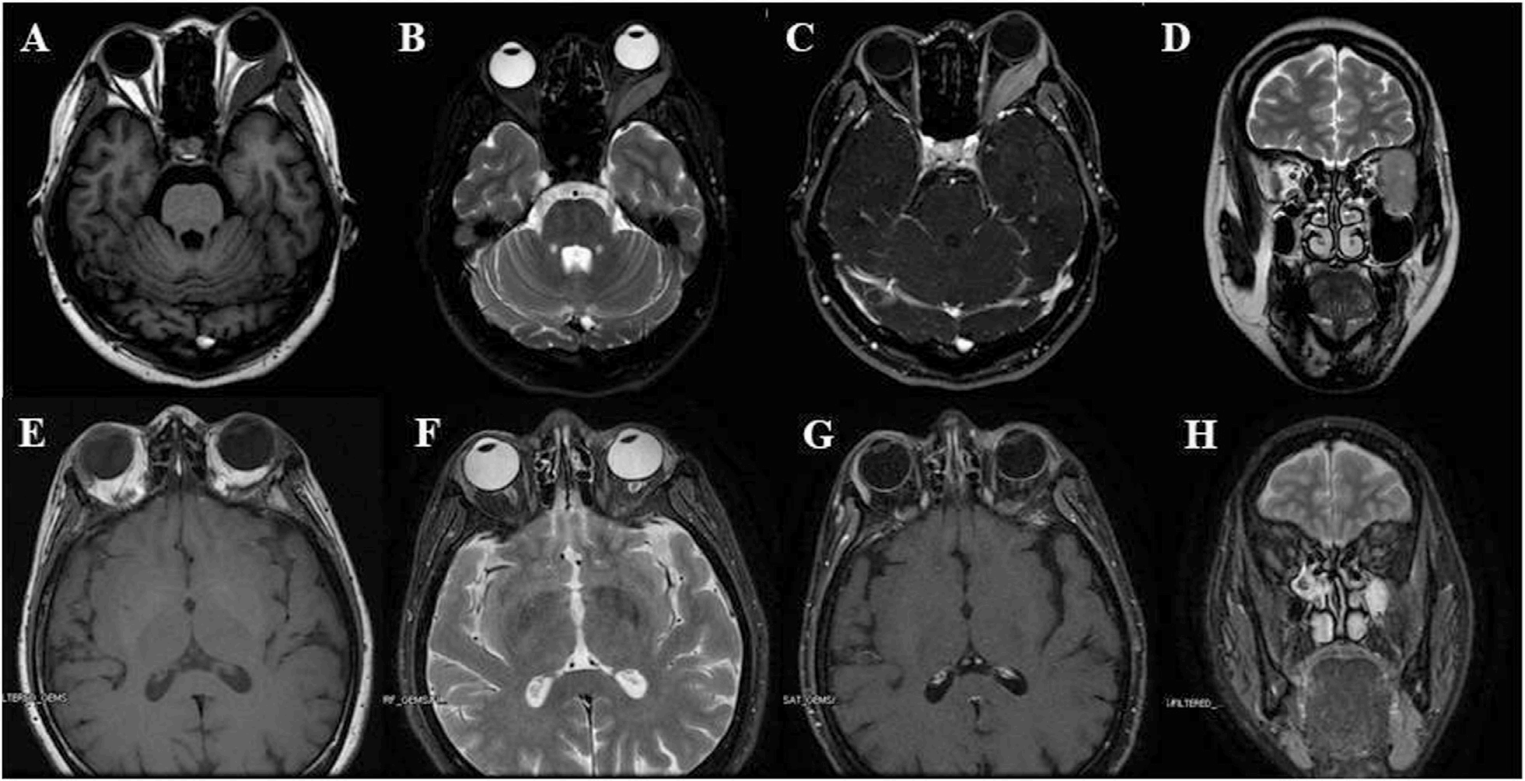
**(A–D)** Pre-operational orbital MRI showing T1W **(A)**, fat-suppressed, axial T2W **(B)**, fat-suppressed, strengthen scanning, axial T1W **(C)** and coronal T2W **(D)** images, it presented a well-defined expansive mass with low T1 and slightly high T2 signals, and pushed the lateral rectus and optic nerve; **(E–H)** Post-operational orbital MRI showing T1W **(E)**, fat-suppressed, axial T2W **(F)**, fat-suppressed, strengthen scanning, axial T1W **(G)** and coronal T2W **(H)** images: no nodule or mass was observed in left orbit.

After sustaining an elbow injury in 2016, a 33-year-old Chinese female experienced anterior proptosis in her left eye, a condition that persisted for 3 months. On examination, the left eye was protruding (see Figure 1A), the inner lower conjunctiva was congested, edematous, and exposed (see Figure 1B), the surface was partially keratinized, the left eye was incompletely closed, all directions of ocular motility were restricted, the pupil was slightly dilated compared to the right, the light reflex was blunted, and no important abnormalities were seen in the fundus of either eye. The patient had a best-corrected vision of 20/17 OD and 20/200 OS. Eye pressure in both eyes was normal. On admission, her peripheral blood count showed hemoglobin (Hb) of 140 g/L, platelets (PLT) of 244 × 10^9^/L, white blood cells (WBC) of 7.72 × 10^9^/L, neutrophils of 3.75 × 10^9^/L, monocytes of 0.39 × 10^9^/L, and slightly higher lymphocytes of 3.47 × 10^9^/L. CT imaging revealed (see Figures 1C–D) irregular soft tissue occupancy in the external space of the muscle cone of the left eye, spreading posteriorly to the orbital apex and compressing the left eyeball, the external rectus muscle and the optic nerve. The woman was scheduled for excision of the orbital mass. The tumor was located in the lateral and muscular cone of the left orbit and was grayish-white, hard, brittle, poorly defined 4 cm × 3.5 cm × 1 cm in size, pale yellow in section, solid and tough (see Figure 1E). The patient had tumor cells expressing LCA, MPO, CD43, and CD117 and no obvious tumor cell infiltration on bone marrow cytology (see Figure 2). The pathological diagnosis was orbital MS of the left eye. The patient subsequently underwent AML-like chemotherapy consisting of standard-dose cytarabine (Ara-C) for seven consecutive days in combination with daunorubicin for 3 days, followed by medium-to high-dose Ara-C in combination with idarubicin after complete remission (CR), as well as local radiotherapy and allogeneic hematopoietic stem cell transplantation (allo-HSCT). There were no signs of recurrence 4 years after surgery (see Figure 3).

### Case 2

**FIGURE 4:**
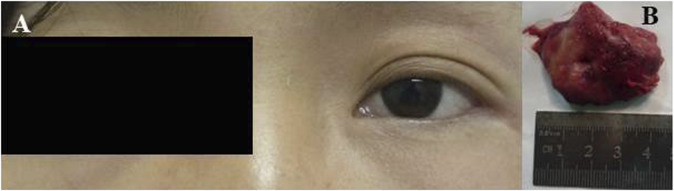
**(A)** Left preoptic bulge. **(B)** Orbital mass resected from patient 2.

**FIGURE 5:**
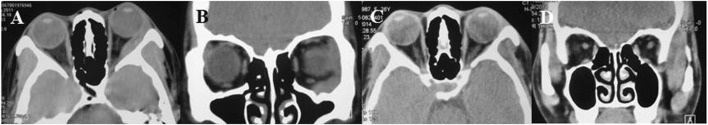
**(A, B)** Pre-operative axial and coronal CT view showing tumour in the left orbit, engulfing external inferior rectus muscle, **(C, D)** 3 years post-operative CT view showing no nodules or masses in the left orbit and eye.

**FIGURE 6:**
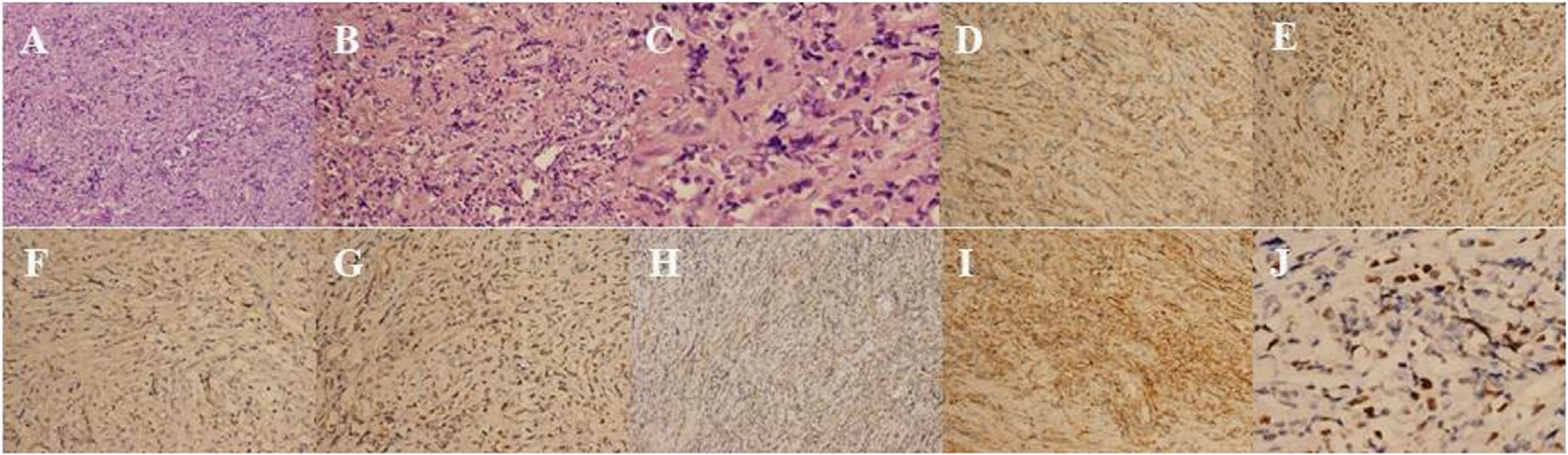
**(A)** HE, hematoxylin–eosin stain × 50; **(B)** HE, hematoxylin–eosin stain × 100; **(C)** HE, hematoxylin–eosin stain × 200; **(D)** LCA, Leukocyte Common Antigen × 100; **(E)** MPO, myeloperoxidase positivity × 100; **(F)** CD99 × 100; **(G)** bcl-2 × 10; **(H)** CD43 × 50; **(I)** CD34 × 100; **(J)** Ki-67 × 200.

A 24-year-old Chinese girl requested treatment in 2012 for left eye prominence that had been present for 4 months (see Figure 4A). Her corrected visual acuity was 20/20 OU. Swelling along the orbital rim was palpable in the lacrimal area of the left eye and lower lid, medium in quality and not pushable. The right eye had a proptosis of 13 mm and the left eye had a proptosis of 15.5 mm. Both eyes had regular anterior segments on fundus examination and no critical abnormalities. Laboratory tests revealed the following data: hemoglobin (Hb), 112 g/L; platelet (PLT) count, 198 × 10^9^/L; white blood cell (WBC) count, 5.71 × 10^9^/L; neutrophil count, 3.94 × 10^9^/L; lymphocyte count, 1.45 × 10^9^/L; and monocyte count, 0.27 × 10^9^/L. A CT scan revealed (see Figures 5A, B) soft tissue shadowing in the inferior part of the left orbit and the left inferior rectus muscle was pushed into the inferior temporal fossa along the lateral wall of the orbit. The postoperative pathological specimen from the patient was a grayish gray-brown unshaped tissue, 4.5 cm × 3.5 cm × 1 cm in size, that appeared to be partially enveloped, with a grayish-white, solid to hard surface (see Figure 4B). The girl showed diffuse infiltration of tumor cells with scattered eosinophils, large, round or oval tumor cells with irregular arrangement, ovoid nuclei, apparent nucleoli, and lack of cytoplasm. Immunohistochemistry showed positivity for LCA, MPO, CD99, bcl2, CD43, CD34 and a Ki67 index of approximately 40%, IgH gene rearrangement (PCR method) with clonal rearrangement bands was detected (see Figure 6). The pathological diagnosis was orbital MS of the left eye. The patient subsequently underwent AML-like chemotherapy (standard dose Ara-C for seven consecutive days, concomitant with doxorubicin for 3 days, followed by high-dose Ara-C after CR) and local radiotherapy and showed no signs of recurrence 10 years after surgery (see Figures 5C, D).

## Discussion

Known also as granulocytic sarcomas (GS) or chloromas, MS is a neoplastic mass of myeloid blasts, with or without maturation, occurring at an anatomical site other than the bone marrow infiltration of leukemia cells and where the tissue architecture is affected. The 5th edition of the World Health Organization Classification of Haematolymphoid Tumours (WHO-HAEM5) states that MS represents a unique tissue-based manifestation of AML, transformed myelodysplastic neoplasm (MDS), myeloproliferative neoplasm (MPN) or MDS/MPN [1]. More specifically, MS is more often seen as a specific manifestation of AML. On the other side, MS is classified as leukemic MS (AML with extramedullary infiltration) or non-leukemic MS/ isolated MS (IMS) [3]. Within 30 days of the diagnosis of MS, bone marrow smears and biopsies are negative, and physical examination, imaging, and laboratory tests confirm that the extramedullary region is the only site of the tumor. We diagnosed it as IMS. It is clinically rare, accounting for 1%–2% of MS.

Orbital MS is mainly seen in the pediatric population. In adults, the most common sites of development of MS are the soft/connective tissues, skin, and gastrointestinal system, with rare occurrences in the orbits [3]. The most common clinical manifestations of orbital MS are protruding eyeballs and orbital masses, with some patients presenting with decreased visual acuity, eyelid swelling, ocular dyskinesia, diplopia, conjunctival congestion, and edema. It has been reported in the literature that on CT, orbital MS appears as a well-defined mass that is isointense or dense with brain tissue and enhances uniformly after contrast injection. On MRI, the orbital lesion shows an isosignal or slightly hypointense signal on both T1WI and T2WI. On enhancement scans, homogeneous enhancement may be demonstrated [5]. For cumulative ocular MS, MRI provides more comprehensive and helpful disease information than CT [6].

Usually, MS can be diagnosed quickly in patients who have had AML, whereas diagnosing IMS can be challenging because it does not involve the bone marrow or other sites and relies heavily on obtaining biopsy specimens for pathology and immunohistochemistry to confirm the diagnosis. Even with adequate biopsy specimens, pathological evaluation of MS is not entirely accurate, and approximately 40%–47% of MS is misdiagnosed, most commonly in diffuse large B-cell lymphoma [3, 7]. MS often expresses MPO, CD43, CD68, and CD117 [8]. Our pathological resection specimens showed that under the light microscope, the tumor cells were diffusely distributed, the cells were medium-sized with small and lightly stained cytoplasm and oval or round nuclei, and the nucleolus was scattered with juvenile eosinophils. Using immunohistochemistry, we found that the tumor cells were positive for LCA, MPO, and CD43, which confirmed orbital MS. In Case 2, we observed the phenomena of IgH gene rearrangement and bcl-2 overexpression, which offered a unique perspective. Traditionally, IgH rearrangement has been considered a hallmark feature of B-cell tumors, involving the aberrant juxtaposition of IgH enhancers with oncogenes (such as bcl-2), resulting in overexpression and activation of these oncogenes. However, a study has reported, for the first time, two cases of myeloid tumors that also exhibited IgH rearrangements, suggesting that oncogenic mechanisms specific to B-cell tumors may also be operative in myeloid tumors, albeit with activation pathways that require further investigation [9].

There are no randomized trials of optimal treatment for patients with MS. Both multiple sclerosis with bone marrow involvement and IMS require systemic therapy for the underlying leukemia [10]. The current standard treatment for AML is Ara-C + anthracycline (7+3) as the first-line remission treatment. After achieving CR, HSCT or Ara-C-based consolidation chemotherapy was used as post-remission therapy. In addition, various small-molecule-targeted and immune-targeted new drugs for AML are being developed and gradually used in the clinic [11]. Allo-HSCT is an effective treatment for MS [10]. Chevallier et al. evaluated the prognosis of 99 MS patients who underwent allo-HSCT, among whom 30 were IMS patients and 69 were MS patients with AML. The 5-year survival rates of the two groups were 48% and 36%, respectively, with no statistically significant difference. However, allo-HSCT after CR could prolong the leukemia-free survival [12]. MS is very sensitive to ionizing radiation [13]. When extramedullary progression or bone marrow recurrence occurs, local radiotherapy is recommended for reasonable control of the disease in the local area and symptom relief without notable toxicity [14].

Interestingly, in Case 1, there was a history of orbital trauma with clinical manifestations of ocular prominence, ocular redness and ocular distention, which could easily be misdiagnosed, and the patient was treated conservatively in other hospitals for 2 months without improvement and with progressive loss of vision. The diagnosis was made definitively only after resection of the mass to determine the cause and obtain a biopsy specimen for pathological histology. In clinical practice, if a patient has a history of ocular trauma causing proptosis that does not improve with nonsurgical treatment, a prompt surgical biopsy is recommended for early diagnosis and treatment. In addition, a history of orbital trauma prior to onset may be the trigger for orbital MS. However, this hypothesis needs to be further investigated.

Some scholars believe that ocular infiltration is a manifestation of the poor prognosis of MS. Zimmerman et al. reported 33 cases of ocular MS, and 10 patients died within 5 months after ocular symptoms appeared [15]. Similarly, Cavdar et al. retrospective analyses of 121 children with AML found that approximately 30% of the children had affected eyes, and the average survival time was only 8.7 months compared to 28.6 months for patients without AML [16]. Paradoxically, Bidar et al. counted 21 cases of orbital MS, and the average survival time reached 6.5 years. In contrast to the previous study, most of Bidar’s patients had a history of leukemia before the onset of ocular disease, which should be considered one of the reasons for the difference [17]. The characteristics of our cases are that both patients had IMS, and there was no recurrence after many years of follow-up. The possible reason is that the period from the onset of the disease to the visit to the doctor was short, and there was no evidence of leukemia. Compared with the previous IMS, the treatment is relatively comprehensive and advanced, and the patients underwent surgery, systemic chemotherapy, and local radiotherapy.

Upon reviewing the course of our study, while we have gained unique insights into the clinicopathological features of orbital medullary sarcoma, the absence of chromosomal and genetic test results, along with limited immunohistochemical details in our case, has hindered our complete comprehension of the disease’s underlying mechanisms. Detecting cytogenetic and molecular biological abnormalities in MS is crucial for both diagnosis and treatment strategies. However, given the rarity of this disease, further research endeavors are imperative.

In summary, MS is a rare disease, and if left untreated, it will rapidly progress to AML in most patients; it is easily misdiagnosed and missed due to the lack of characteristic clinical manifestations. Therefore, timely diagnosis of MS is the key to clinical management, and the diagnosis is confirmed by pathology and immunohistochemistry. Once diagnosed, AML-like chemotherapy regimens should be given as early as possible to improve survival time, with local surgical resection and radiotherapy for symptomatic relief, and early allo-HSCT is recommended if available.

## Data Availability

The datasets presented in this study can be found in online repositories. The names of the repository/repositories and accession number(s) can be found in the article/supplementary material.
